# Case Report: Surgical management of thoracic chondrodysplasia in children: complete mobile thoracic replacement

**DOI:** 10.3389/fsurg.2026.1725855

**Published:** 2026-07-03

**Authors:** Lucie Louis, Luke Harper, Jean-David Maes, Frédéric Lavrand

**Affiliations:** 1 CHU de Bordeaux, Service de Chirurgie Pédiatrique, Bordeaux, France; 2 CHU de Bordeaux, Service D’Imagerie Cardiaque et Thoracique, Pessac, France

**Keywords:** children, Jeune syndrome, sensenbrenner syndrome, surgical management, thoracic dystrophy

## Abstract

**Objectives:**

Several syndromes, including Jeune's syndrome and Sensenbrenner's syndrome, can cause thoracic chondrodysplasia in children, leading to various degrees of thoracic insufficiency syndrome. Surgical management if often necessary and numerous surgical techniques have been described in the past, but all of these offer rigid solutions, which are incompatible with growth, and full recovery of ventilatory dynamics. We describe a new surgical technique for these patients, which offers a complete mobile thoracic replacement.

**Methods:**

We present the case of two patients with Jeune and Sensenbrenner syndromes in whom we performed a complete thoracic replacement. We used the Multidirectional Thoracic Wall Stabilization (MTS) system, (StraTos™ MedXpert's), in order to recreate a mobile thorax, allow for recovery of ventilatory dynamics and enable thoracic growth.

**Results:**

Both surgeries were successful. Long-term follow-up (>4 years), shows both patients regained respiratory autonomy for acts of daily routine, increased their walking perimeter and increased their lung volumes.

**Conclusion:**

Total thoracic replacement is safe and feasible for thoracic dysplasia in children. Further experience is needed to formally compare outcomes with the traditional techniques.

## Introduction

Ciliopathies comprise a heterogeneous group of genetic disorders caused by structural or functional disruption of cilia, or by abnormal cilia biogenesis, leading to a wide variety of phenotypes and clinical manifestations (1). They include Jeune syndrome and Sensenbrenner syndrome, which have a dominant skeletal system involvement, leading to thoracic chondrodysplasia.

Jeune syndrome or asphyxiant thoracic dysplasia is characterized by osteochondrodysplasia. Its incidence ranges from 1 in 100,000 to 1 in 130,000 live births and its transmission is autosomal recessive (2). Its phenotype is widely variable including: lethal, severe, moderate and latent forms (2). The dominant phenotype is characterized by short limbs and a narrow thorax with short ribs. The resulting consequences on respiratory mechanics determine the resulting respiratory impairment, which in itself conditions the vital postnatal prognosis. Depending on how long the child lives, several organs (liver, kidney, pancreas, retina) may also be progressively affected (3–5).

Sensenbrenner syndrome or cranioectodermal dysplasia was first described in 1975 by Sensenbrenner in two siblings (6). It is inherited in an autosomal recessive fashion and is characterized by skeletal damage, including short limbs and a narrow thorax with short ribs, as well as ectodermal damage, which can include widely spaced hypoplastic teeth, sparse hair and abnormal nails. Characteristic facial features are also present, notably dolichocephaly. These children will also suffer from renal failure, hepatic fibrosis and retinal anomalies (7).

In recent years, several surgical procedures have been developed to correct the thoracic anomalies associated with these conditions. These techniques consist mainly in enlarging the rib cage. Some require sternotomies to achieve sternal spacing by internal or external distractor (8–11), sometimes with the aid of plate interposition (12, 13), autologous (14) or homologous (15) bone grafting. Others require rib osteotomies to enlarge the thorax laterally (16–18) or vertically (19). All these techniques are rigid and do not allow the thoracic cage to grow as the child grows, nor do they restore ventilatory dynamics. The results of these techniques are therefore transient, and several interventions will be necessary as the child grows.

We therefore sought to develop a dynamic surgical technique for these children, by resecting all dystrophic parts to create a complete replacement of the rib cage. The aim is to create a set-up that is adapted to the child's growth and enables restoration of ventilatory mechanics. We report our experience with this new technique.

## Methods

We report the cases of two patients managed for thoracic chondrodysplasia in our department of pediatric Surgery. For use of patient data for this study, we obtained written consent from the parents of both children as well as written consent from the eldest of the two children.

Following our experience using the MedXpert’s StraTos™ systems (Strasbourg Thorax-Osteosynthese-System) and StraCos™ (Strasbourg Costale-Osteosynthese-System), for chest wall and/or sternum reconstruction after the management of tumors or thoracic pathologies requiring the resection of affected tissue or bone, we decided to try this material for children with thoracic dysplasia.

**Figure 1:**
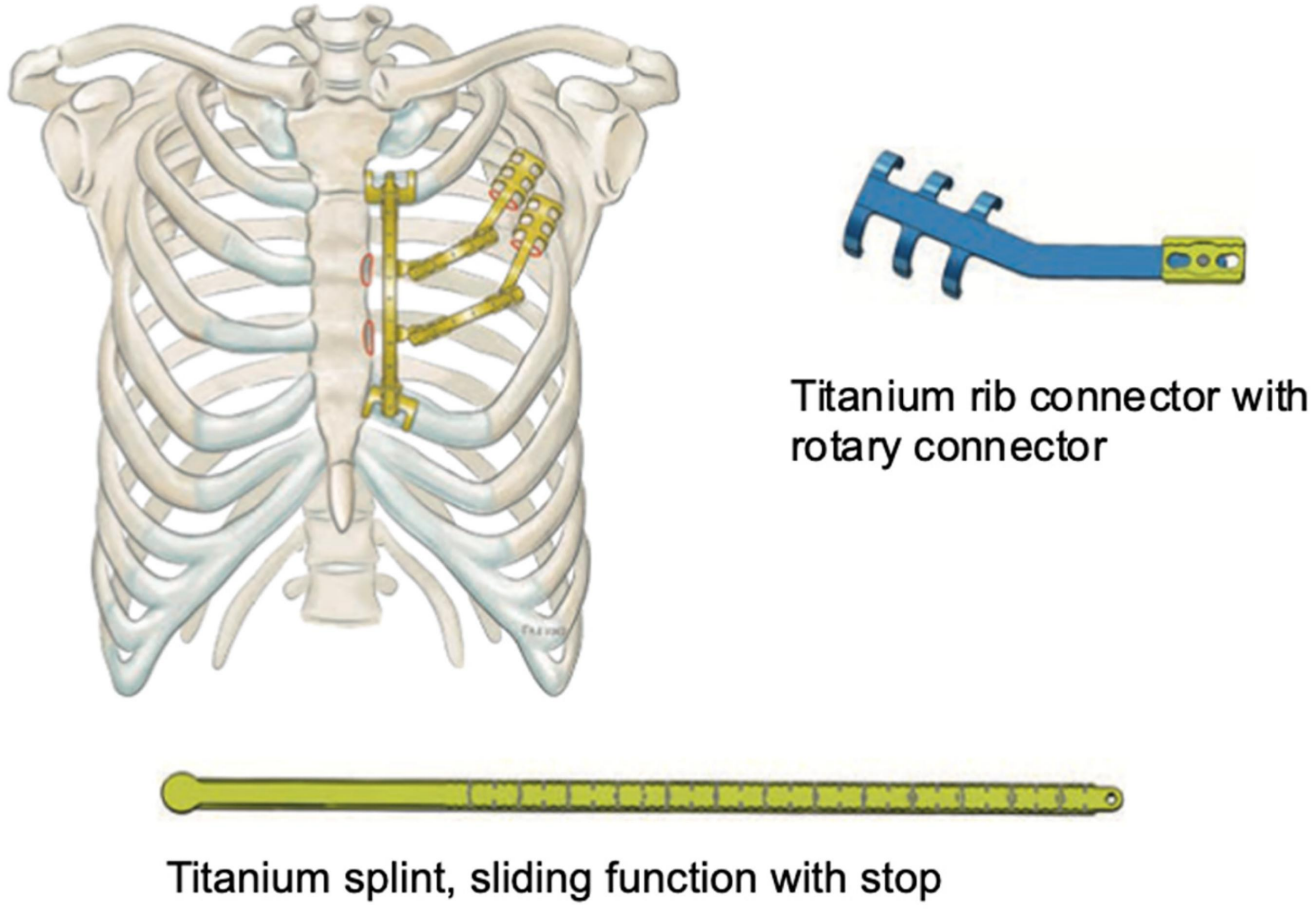
Multidirectional thoracic wall stabilization (MTS) system, Source: MedXpert's straTos_TM_, https://medxpert.de/.

More specifically, the Multidirectional Thoracic Wall Stabilization (MTS) system is equipped with connectors on which are rotatable connectors (swivels) into which the splints can be inserted. These ball-and-socket joints enable the system to move in all directions. The unique feature of this system is that we have fitted it with a splint featuring a release mechanism on one side, allowing it to slide into the rib connector, thus permitting growth in all planes, without the risk of the splint becoming detached from the connector. This has enabled us to adapt the device to accommodate growth, thereby allowing it to be used in children (Figure 1).

### Surgical technique for complete chest wall replacement using the multidirectional thoracic wall stabilization (MTS) system

The procedure begins with parietal resection performed through a Clamshell-type anterior thoracic herringbone incision. The entire costal arches and corresponding chondrosternal dysmorphic regions (generally the 3rd to 7th ribs) are then resected. Dissection is subperichondral and extrapleural. Laterally, rib section is usually performed at the posteromedial junction, where the rib axis is subnormal. The intercostal muscles and pedicles are left intact. After parietal resection, lung re-expansion can be noted visually, as well as an increase in volumes on ventilator parameters.

The second stage consists in reconstructing the chest wall as anatomically as possible. As the assembly is made to measure on a case-by-case basis, our technique varies according to the child's size.

**Figure 2:**
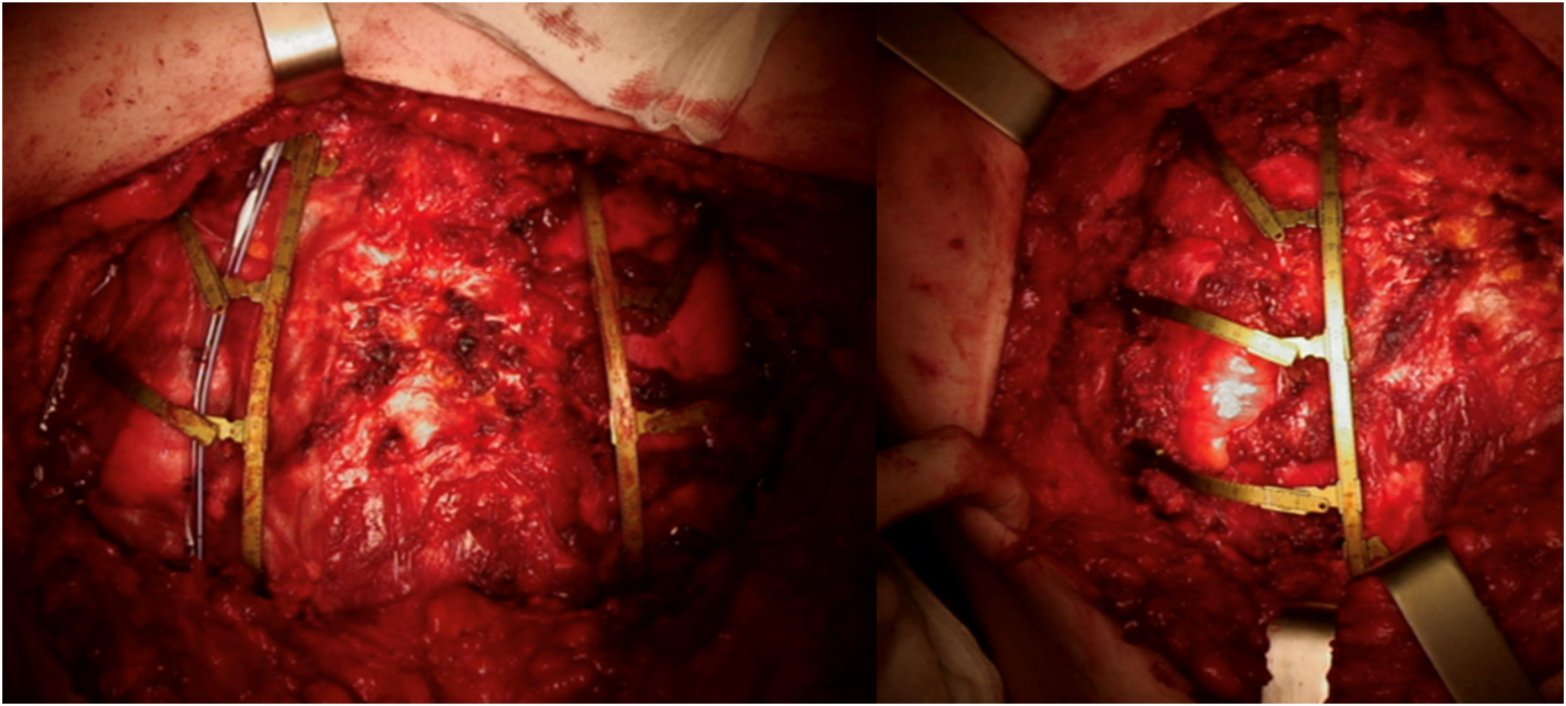
Complete chest wall replacement using a multidirectional thoracic wall stabilization (MTS) system in children with a large thorax, intraoperative view.

For children with a large thorax (as in our first case), the first step is to fit a parasternal splint, which is attached to the chondrosternal cartilages of the 3rd and 8th ribs by means of upper and lower ball-jointed tripod connectors. It should be noted that 3 articulated connectors are placed on the splint before it is fixed. This is followed by the placement of ball-and-socket joints, to replace every second costal level, in the lateral costal section. Finally, splints are placed horizontally between the costal and para-sternal connectors, to replace the costal arches. These splints are placed in a highly distracted and rounded position, with the aim of reconstructing the ribcage as anatomically as possible (Figure 2). Once thoracic reconstruction is complete, we apply Vicryl® plates to cover the muscle masses, which are then partially attached to the splints. In our first case, we locked all the splints from the outset, as the patient was in late growth and the rib cage had been oversized at the time of fitting. The main aim was to restore ventilatory dynamics. All connections and clasps are ball-jointed, making the system completely mobile as the ribcage expands.

**Figure 3:**
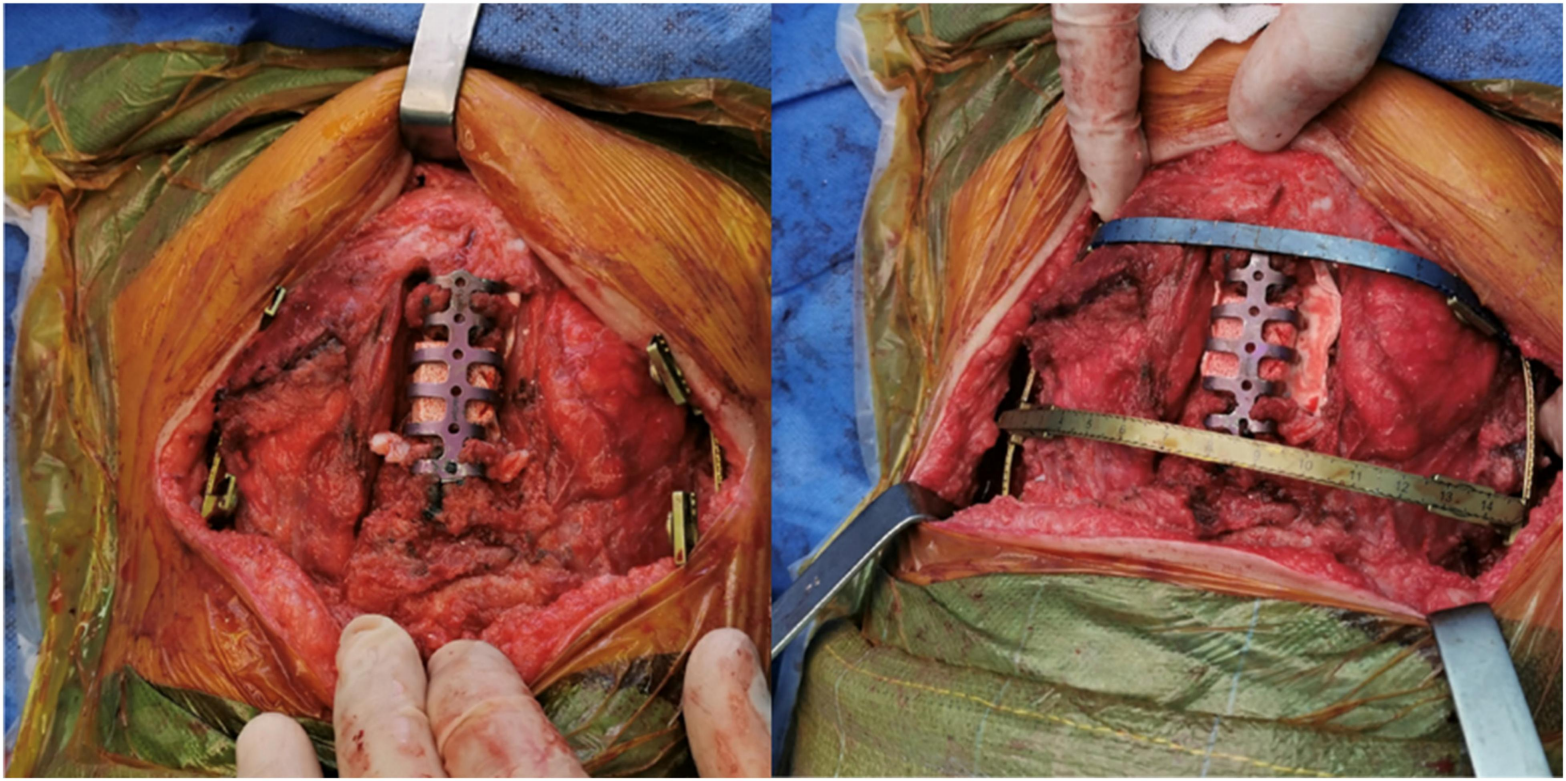
Complete chest wall replacement using a multidirectional thoracic wall stabilization (MTS) system in children with a small thorax, intraoperative view.

For children with a small thorax (as in our second case), reconstruction begins with the placement of a vertical arched splint on the right and left sides of the thorax, which is attached to the remaining upper and lower ribs using a tripod ball-and-socket joint. T-connectors, also ball-jointed, are fitted to both splints. The brace is then locked to the tripod connectors at the top and bottom. To complete the enlargement, a sternal “split” is performed by cutting the sternum transversely, then after spreading the two ends, a bone substitute is inserted over a length of approximately 2 cm. This is secured to the sternum with a StraCos™ splint, after a GORE-TEX® plate has been placed behind it to protect the pericardium. We then proceed to finalize the set-up by interposing two StraTos™ splints in front, which are attached to the lateral mobile T-connectors, and bridge the entire ribcage in front of the sternal reconstruction (Figure 3). Two Integra® collagen matrix reinforcements are placed in front of each lung to protect the lungs and create a gliding plane. It should be noted that, in the case of an infant with a very narrow thorax that does not allow longer splints to be fitted for growth purposes, the set-up had to be locked. In a second phase, however, the central part of the chest can be enlarged by the addition of ball-and-socket joints, into which non-locking sliding splints can be inserted to allow the ribcage to grow.

## Case presentation

1. Case 1: complete rib cage replacement in a child with a large thorax
  The first patient presented with a post-natal diagnosis of Sensenbrenner syndrome. He showed good adaptation at birth, and good respiratory evolution in the first years of life. The disease was characterized by repeated episodes of severe pneumopathy requiring hospitalization in intensive care. Initially, he required ventilatory assistance with nocturnal NIV, then became dependent on oxygen therapy during the day, with the need to use a wheelchair. His evolution was marked by numerous episodes of pulmonary infections, necessitating alternate antibiotic therapy. We therefore decided to perform a complete rib cage replacement at the age of 12.
2. Case 2: complete rib cage replacement in a child with a small thorax
  The second patient presented with an antenatal diagnosis of Jeune's syndrome. He required non-invasive ventilation (NIV) at 10 min of life. During the next few days, he showed progressive respiratory deterioration with respiratory acidosis necessitating intubation on the 8th day of life, with no weaning possible.

In view of the severity of the clinical picture, it was decided to perform neonatal salvage surgery, using sternochondroplasty to enlarge the sternum at 6 weeks of age thus allowing adequate breathing. The procedure involved parietal resection and the insertion of two overlapping sternal splints, held in place by a ball-and-socket joint. This central patella offered the possibility of a few degrees of mobility. This set-up offered a transitional solution, with the possibility of thoracic expansion corresponding to around three times that previously available. In the immediate post-operative period, the patient required invasive ventilation for eleven days. Subsequently, given the impossibility of weaning the patient off invasive ventilation, it was decided to perform a tracheotomy two months post-operatively, which enabled the patient to be ventilated on a home ventilator with the possibility of a spontaneous breathing period (15 min maximum).

At the age of 17 months, the patient presented a worsening respiratory condition with equipment that had become too small, resulting in respiratory insufficiency and the need for permanent respiratory assistance with major difficulty in maintaining ventilatory parameters. We decided to carry out a complete chest wall replacement, this time using a mobile system that could be modified for growth in an infant.

In this much younger patient, we did not have sufficient space to allow reconstruction of two hemithoraxes. We therefore decided to bypass the thoracic cavity, using mobile splints on a patella to allow dynamic ventilation. However, the narrowness of the thoracic cavity prevented us from using non-locking sliding splints with a central ball-and-socket joint to allow thoracic growth. The whole set-up had to be locked.

## Results

**Figure 4:**
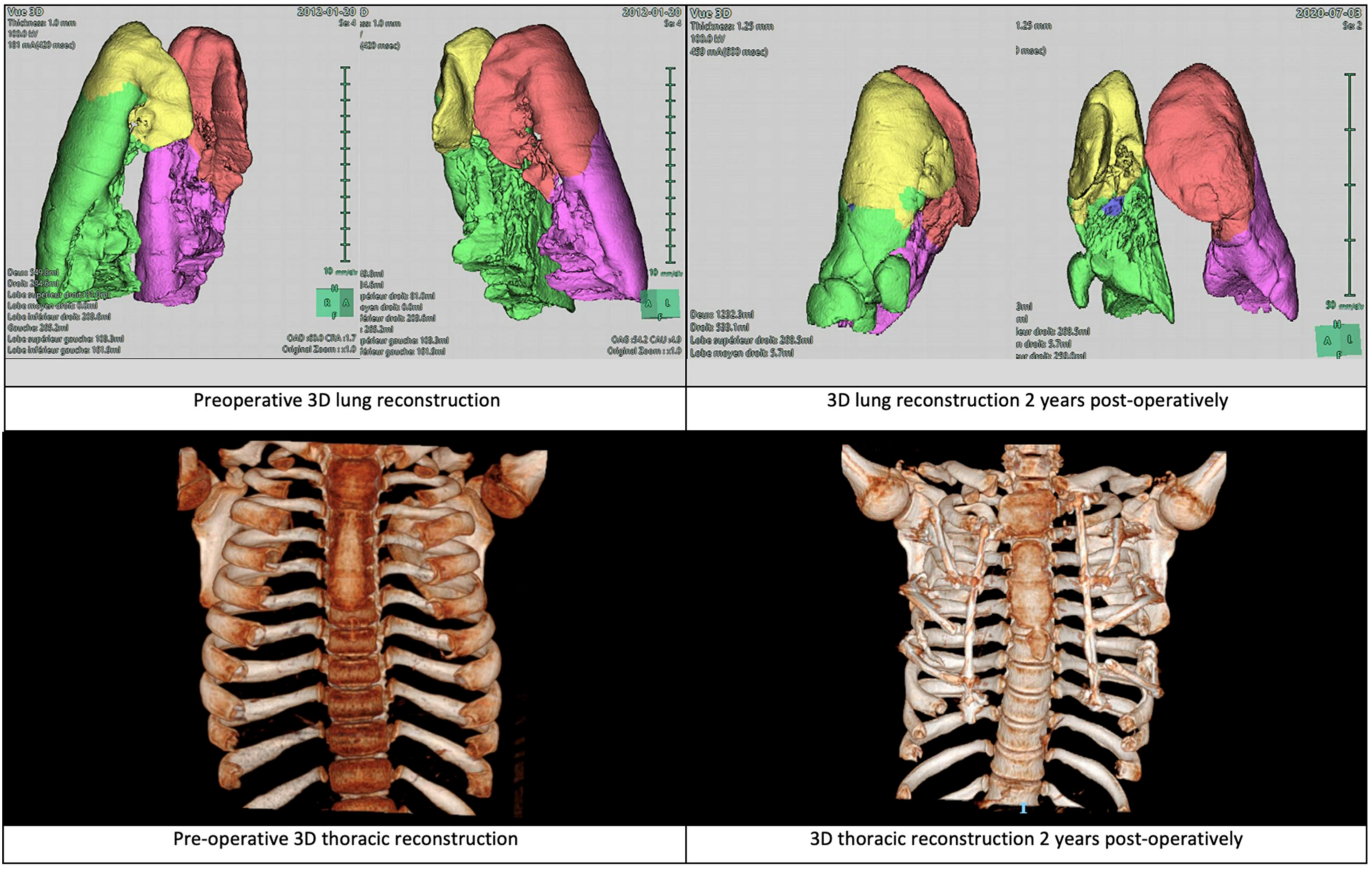
Pre-and post-operative 3D lung and chest reconstruction in children with a large thorax.

Patient 1: Postoperatively, there was a progressive improvement in respiratory function, with an increase in daytime NIV-free periods with oxygen therapy alone. At one year postoperatively, the patient was able to walk up to 150 meters and was able to breathe independently during daily activities without oxygen therapy. He required only nocturnal non-invasive ventilation. Room air saturation had risen to 97%, compared with 85% on 2L of oxygen preoperatively. Subsequently, complete cessation of daytime oxygen therapy with full respiratory autonomy for daily activities, cessation of wheelchair use with independent movement. We observed an increase in lung volumes in the post-operative period (Table 1). No respiratory decompensation or infectious episode (Figure 4). At the latest follow-up, at 6 years post-operatively, the patient presented no complications and was able to return to school.

**Table 1:** CT measurement of lung volumes before and after surgery in patient 1.

TIME POINT	TOTAL LUNG VOLUME (CM^3^)	AGE-PREDICTED NORMAL LUNG VOLUME (CM^3^) *	RIGHT LUNG VOLUME	LEFT LUNG VOLUME
**PREOPERATIVE (AGE 7 YEARS)**	550	1233	285	265
**POSTOPERATIVE (2-YEAR FOLLOW UP, AGE 15 YEARS)**	1232	2453	533	699

* Age-predicted normal lung volume using the formula y = (1.27 × 10⁻^2^) x + 0.167 where y is the lung volume in liters (L) and x is the subject age (26).

Patient 2: The patient was extubated on the tenth post-operative day, with resumption of ventilation on his home ventilator via the tracheostomy. Following the operation, there was a marked improvement in respiratory function, with spontaneous breathing gradually increasing from 5 to 7 h a day on 1–2L/min oxygen, with nocturnal ventilatory support maintained. We observed an increase in lung volumes in the post-operative period (Table 2).

**Table 2:** CT measurement of lung volumes before and after surgery in patient 2.

TIME POINT	TOTAL LUNG VOLUME (CM^3^)	AGE-PREDICTED NORMAL LUNG VOLUME (CM^3^)*	Right lung volume	Left lung volume
**Preoperative (Day 4)**	69	179	38	31
**1 Month After Salvage Procedure**	129	217	75	54
**Preoperative (Prior to Total Chest Wall Replacement)**	263	319	148	115
**1 Year After Total Chest Wall Replacement**	495	548	248	247

* Age-predicted normal lung volume using the formula y = (1.27 × 10⁻^2^) x + 0.167 where y is the lung volume in liters (L) and x is the subject age (26)..

**Figure 5:**
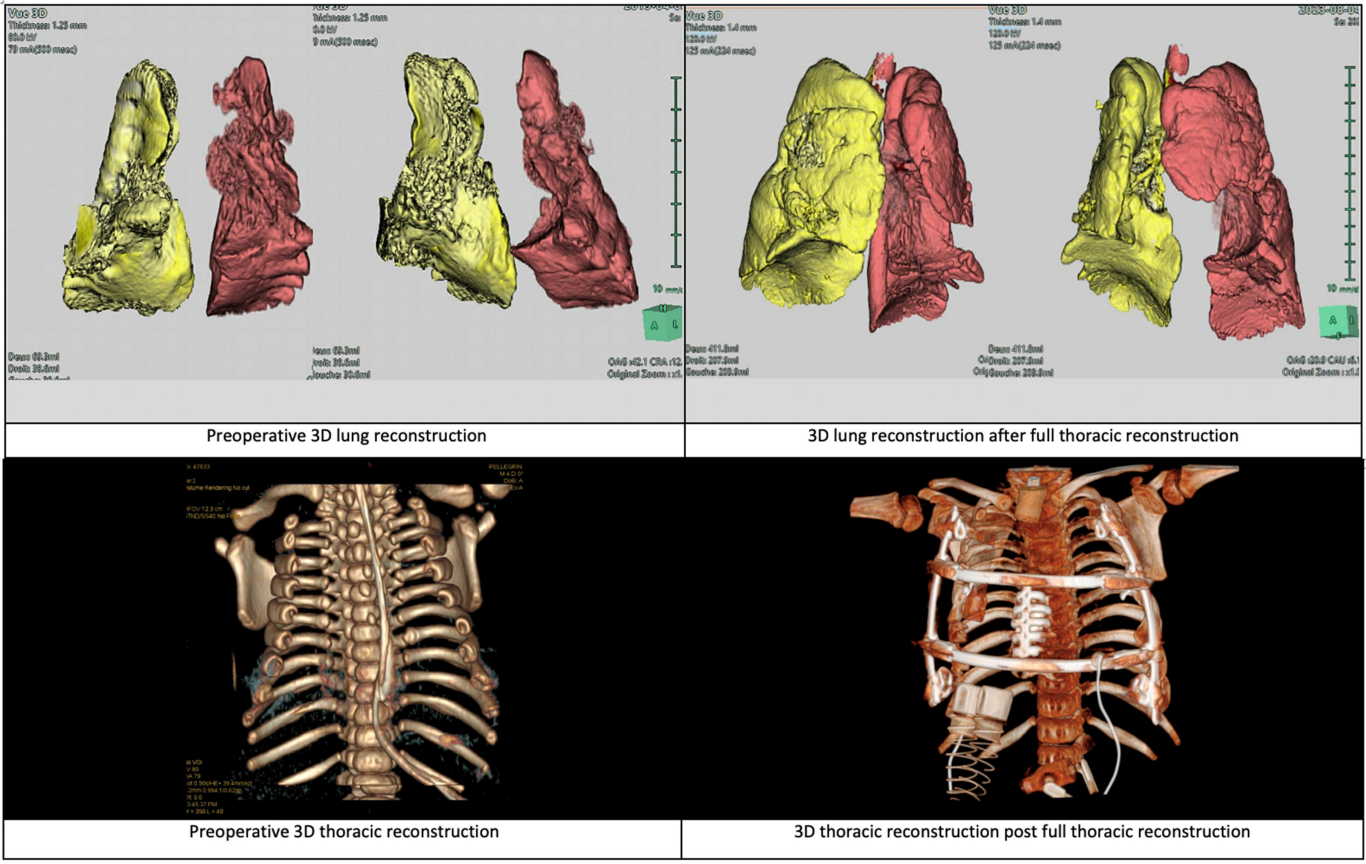
Pre and posT,operative 3D lung and chest reconstruction in children with a small thorax.

At one and a half years post-operatively, only nocturnal and naptime ventilatory support was required, with spontaneous breathing without oxygen during all waking phases. Significant increase in walking and running perimeter (Figure 5). Three years after the operation, a skin irritation developed between the scar and the sternal staple, necessitating its removal.

Currently, at the age of 5 (4 years post-operatively), the patient is on spontaneous ventilation during the day. However, respiratory difficulties have reappeared, with a decrease in walking perimeter and repeated episodes of pneumopathy. We have therefore reached the limit of this set-up and plan to carry out a new operation soon. This will involve the creation of two hemithoraxes, with Case 1 as model, and the difference being the use of non-locking sliding splints to allow his chest wall to grow.

## Discussion

We present two cases of mobile rib cage replacements for severe thoracic dysplasia with satisfactory results. The management of thoracic chondrodysplasias is not standardized, as these are rare diseases that few surgeons will encounter during their practice. There are no guidelines, recommendations or consensus conferences concerning the ideal surgical approach for the management of these pathologies.

The aim of the initial management of these children is to enable adequate breathing despite an uncompliant thorax. Given the great clinical variability of these pathologies, management is also highly heterogeneous, and can take place in the neonatal period for the most deleterious forms, or in childhood following repeated episodes of pneumopathy for less severe forms.

In theory, alveolar development can continue until the age of 8 (20). Since alveolar growth potential is normal in children with thoracic chondrodysplasias, lung changes are secondary to thoracic space insufficiency. One might assume that if the thorax could be enlarged, lung growth would improve lung function. Consequently, the only part of the disease that can theoretically be corrected is thoracic size and mobility (21).

Depending on age and severity, numerous techniques have been developed, all of them experimental. A classification of these techniques has recently been carried out (22). These techniques differ in terms of invasiveness, material and approach. Most of these techniques have only been proposed and performed in isolated cases, due to the extreme rarity of these syndromes. A common problem with most of these techniques is that they prevent thoracic growth and mobility due to rigid mounting. Although promising medium-term results have been reported, these life-saving expansion thoracoplasties have only served patients for a short time, as they represent a static solution unable to meet the growth requirements of these children (Table 3).

**Table 3:** Advantages and limitations of surgical techniques for Major chest wall reconstruction in pediatric patients.

Surgical Technique	Advantages	Limitations
Prosthetic reconstruction (methyl methacrylate, allograft bone)	Immediate stability; effective for large defects	No growth potential; infection risk; likely need for revision
Progressive distraction devices (external/internal)	Allows thoracic growth; improves pulmonary development; suitable for early childhood	Multiple procedures; device-related complications; high treatment burden
Lateral thoracotomy	Direct access; adaptable for localized defects	Postoperative pain; potential impact on growth; limited for extensive defects
Vertical thoracotomy (VEPTR)	Promotes thoracic growth; increases lung volume; suitable for complex deformities	High complication rate; repeated surgeries; long-term burden
Complete mobile thoracic replacement	Allows thoracic growth; biocompatible and structurally stable material; preserves respiratory dynamics through mobility; restores thoracic morphology and may limit spinal deformities	May require secondary surgery in very young children

We therefore set out to develop a technique for complete rib cage replacement, to restore ventilatory dynamics while allowing the chest wall to grow. This technique represents a paradigm shift from existing techniques involving enlargement of dystrophic, poorly mobile structures. Our technique offers several advantages.

First, it enables resection of all the dystrophic elements of the thoracic cage. This eliminates the internal pull on the thorax created by the dystrophic chondrosternal cartilage, thereby immediately increasing the volume of the ribcage. Secondly, it enables replacement of the thoracic cage with a titanium ball-and-socket joint, thus restoring ventilatory dynamics. Furthermore, the chest wall is reconstructed using only the non-dystrophic ribs for support, since these are dynamically functional, and the intercostal muscular system is preserved. Thirdly, thanks to the sliding splints inside the connectors, the thoracic wall can grow as the child grows, as we have already observed in cases of oncological thoracic reconstruction. Lastly, it enables us to preserve a subnormal thorax morphology, limiting the spinal sequelae that can occur with other techniques.

In addition, this system is solid and reliable for chest wall reconstruction, since we have not observed any breakage to date. It is to be noted that some oncology patients treated with the same technique with a follow-up of more than 10 years. Moreover, the fact that this material is made of titanium is an advantage over other metals, as it produces fewer artifacts than steel, which is important in the follow-up of these patients (23). Titanium is not ferromagnetic, which makes it possible to examine patients by magnetic resonance imaging, which is an advantage in children (24).

There are, however, limitations to our technique. It is technically impossible to dynamize the assembly from the outset in very young children, thus requiring one or more subsequent interventions to allow thoracic growth. This factor must be considered when choosing the technique. Our technique requires implantation of a lifelong foreign material, though this is compensated by the high biocompatibility and flexibility of titanium. Indeed, titanium possesses unique properties, including exceptional mechanical and corrosion resistance, as well as biocompatibility (25).

Our experience is limited to two patients, but we believe the promising results we have observed show that the technique is safe and feasible and justifies pursuing this technique. Of course, a larger series is necessary to be able to formally compare the results of this technique with the results of previously described cases. It can be noted that we have long-term follow-up for our cases, which is not always the case in reported series.

## Conclusion

Complete rib cage resection and mobile reconstruction for severe thoracic dysplasia in children is safe and feasible. We believe this technique can be considered for the management of these patients.

## Data Availability

The clinical data is available on specific request to the authors. Requests to access these datasets should be directed to frederic.lavrand@chu-bordeaux.fr.
